# Screening for tuberculosis infection prior to initiation of anti-TNF therapy

**DOI:** 10.1016/j.autrev.2008.07.011

**Published:** 2008-12

**Authors:** Ajit Lalvani, Kerry A. Millington

**Affiliations:** Tuberculosis Immunology Group, Department of Respiratory Medicine, National Heart and Lung Institute, Imperial College London, Norfolk Place, London W2 1PG, UK

**Keywords:** Anti-TNF therapy, *Mycobacterium tuberculosis*, Tuberculin skin test, T-cell interferon-gamma release assays

## Abstract

T-cell interferon-gamma release assays (IGRAs) are more specific and probably more sensitive than the tuberculin skin test (TST) for the diagnosis of latent tuberculosis infection (LTBI). Patients with immune-mediated inflammatory diseases (IMID) and suspected LTBI who are candidates for anti-TNF therapy are at a significant risk of TB reactivation yet are prone to false-negative TST results because they are already on immunosuppressive medications. The role of these new blood tests in this patient population is therefore of considerable interest but is currently unclear. The limited published evidence-base shows that agreement between IGRA and TST results is poor in patients with IMID compared to patients without IMID, due to lower proportions of TST-positive results in patients with IMID. Discordant TST-positive, IGRA-negative results are associated with prior BCG vaccination and discordant TST-negative, IGRA-positive results are associated with steroid therapy. Notably, positive IGRA results are more closely associated with the presence of risk factors for LTBI than TST. The percentage of indeterminate IGRAs can be up to 12%. IGRA results in patients already taking anti-TNF agents currently remain uninterpretable. Given the clinical imperative to prevent reactivation of TB in patients starting anti-TNF therapy, screening algorithms should maximise diagnostic sensitivity for detection of LTBI. Therefore, a positive result to either an IGRA or TST, in addition to currently recommended clinical screening for risk factors for LTBI, should prompt consideration of preventive treatment of LTBI in this population.

## 1 The risk of incident tuberculosis during anti-TNF therapy

**Fig. 1:**
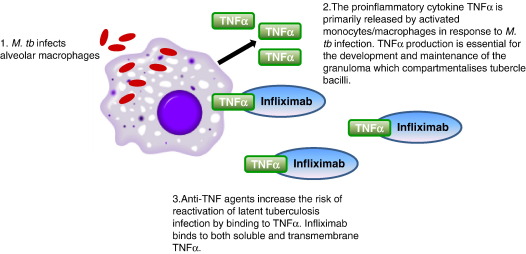
Schematic representation of the essential role of TNFα in host immunity against M. tb infection. In patients latently infected with M. tb the risk of incident tuberculosis is 4 to 5 fold higher after initiation of anti-TNF therapy with infliximab. However, it remains unclear whether the TNFα receptor antagonist, etanercept, or the fully humanised monoclonal antibody, adalimumab, increases the risk of TB above the elevated baseline TB incidence rates already documented for patients with IMIDs.

Therapeutic blockade of tumour necrosis factor alpha (TNF) has emerged as an effective treatment in immune-mediated inflammatory diseases (IMID) such as rheumatoid arthritis, ankylosing spondylitis, Crohn's disease and psoriatic arthritis. However, TNFα is a key cytokine in protective host defence against *Mycobacterium tuberculosis* (*M. tuberculosis*) and, together with TNF-dependent chemokines [34] play an important role in the development and maintenance of the granuloma which compartmentalises tubercle bacilli during infection [35,36] (Fig. 1). Inhibition of TNFα and TNF-regulated chemokine networks is the presumed biological basis for the 4 to 5 fold higher incidence of tuberculosis observed after initiation of anti-TNF therapy with infliximab, in patients latently infected with *M. tuberculosis* [1–3]. It remains unclear though, despite there being case reports, whether the TNFα receptor antagonist, etanercept, or the fully humanised monoclonal antibody, adalimumab, increases the risk of TB above the elevated baseline TB incidence rates already documented for patients with rheumatoid arthritis (RA) [4,5,37]. Development of active TB once infliximab treatment is started is rapid, with a median onset of 12 weeks and 98% of cases occurring within 6 months of initiation of TNF blockade [1]. Moreover, there is a higher incidence of life-threatening extrapulmonary and disseminated disease than among immunocompetent patients. Exclusion of active TB and treatment of latent tuberculosis infection (LTBI) are therefore clinical imperatives prior to starting anti-TNF therapy and active surveillance for a history of untreated or partially treated TB or LTBI has already been shown to be effective in reducing the number of incident TB cases [2,3].

## 2 Current clinical practice

In the absence of a gold standard test for diagnosis of LTBI, current clinical management of patients with IMID requiring anti-TNF therapy involves checking for a history of untreated or partially treated TB, risk-stratification for exposure to cases of active TB, evidence of residual changes indicative of prior TB infection on a chest radiograph and a tuberculin skin test (TST).

The TST is a measure of the delayed-type hypersensitivity reaction to intradermal inoculation of purified protein derivative (PPD), a crude mixture of more than 200 *M. tuberculosis* proteins. Because antigens within PPD are also found in other mycobacteria, the TST suffers from poor specificity in bacille Calmette–Guérin (BCG)-vaccinated persons. Moreover, the sensitivity of the tuberculin skin test used to diagnose LTBI is compromised in patients on immunosuppressive therapy with a high rate of false-negative TST test results. For example, in a Peruvian study, the size of the TST response was significantly lower and the proportion negative (i.e. < 5 mm) to TST was significantly higher in patients with RA compared to healthy immunocompetent controls (median size PPD induration 4.5 vs 11.5 mm *P* < 0.01; 79/112 (70.6%) vs 25/96 (26%) *P* < 0.01, respectively) [6]. Logistical hurdles, including the need for a return visit to read the result of this in vivo test and operator variability in inoculation and reading of the result, also limit the effective use of TST.

Thus because almost all patients awaiting initiation of anti-TNF therapy are on immunosuppressive therapy [1], the TST is not an appropriate test to screen for LTBI in patients with chronic inflammatory diseases. This concern around the interpretation of the TST in patients on disease modifying antirheumatic drugs (DMARDs) is reflected in British Thoracic Society guidelines from 2005 that recommended replacing TST with an individual risk–benefit calculation in patients with a normal chest radiograph balancing the population risk of LTBI in different groups based on age and ethnicity with the risk of serious hepatotoxicity secondary to isoniazid therapy [7].

## 3 T-cell interferon-gamma release assays

T-cell interferon-gamma release assays (IGRAs) have been developed as an alternative immunodiagnostic approach to the TST for detecting *M. tuberculosis* infection [8–11]. Two assay formats are used to detect ex vivo either the frequency of pre-sensitised *M. tuberculosis*-specific T cells releasing interferon-gamma (IFN-γ) isolated from peripheral blood mononuclear cells (ELISpot) or the amount of IFN-γ released in whole blood (ELISA) in response to the immunodominant secreted proteins early secretory antigen target-6 (ESAT-6) and culture filtrate protein 10 (CFP-10). The ELISpot assay developed by Lalvani [8,12–16] is commercially available as T-SPOT™.*TB* (Oxford Immunotec, Abingdon, U.K.), and the ELISA is commercially available as either QuantiFERON™-TB Gold (QFT-G, Cellestis, Carnegie, Australia) or QuantiFERON™-TB Gold in-tube (QFT-IT, Cellestis, Carnegie, Australia) which has the additional antigen TB7.7 encoded by a phage-inserted region, RD11.

The antigens used in IGRAs are absent from BCG [17] and most environmental mycobacteria (except *M. kansasii*, *M. szulgai*, *M. marinum*, *M. flavescens* and *M. gastrii*) [18]. Thus T-cell responses to these antigens are not confounded by prior BCG vaccination and are a more specific immune marker of *M. tuberculosis* infection than TST. Moreover, IGRAs are probably more sensitive than TST for diagnosing LTBI. If they prove to be more sensitive in patients with IMID who are prone to false-negative TST results and who are candidates for immunosuppressive medications that increase the risk of TB reactivation they will have high clinical utility in routine rheumatological practice. Here we review the evidence-base to date on the performance of IGRAs in these patients.

## 4 Clinical performance of IGRAs in patients with IMID before anti-TNF therapy

Published data on IGRA performance in the diagnosis of LTBI in IMID is scarce but expanding rapidly (Table 1). IGRAs performance in patients with IMID has been based on agreement of results with the TST and on the relative strength of the association of TST and IGRA results with risk factors for LTBI. The former type of study design is less useful than the latter. Data from studies that is not correlated with surrogate markers of LTBI collectively concludes that (a) agreement between TST and IGRA is poor and weaker in patients with IMID than in healthy controls due to lower proportions of TST-positive results in patients with IMID [19–24], (b) the magnitude of the TST response is significantly lower in patients with IMID than in healthy controls [22], (c) that discordant TST-positive, IGRA-negative results are associated with prior BCG vaccination [24] and that (d) discordant TST-negative, IGRA-positive results are associated with steroid therapy [24].

Only two studies to date have correlated IGRA and TST results with risk factors for LTBI. In 142 patients with IMID, QuantiFERON^®^-TB Gold in-tube was significantly more closely associated with the presence of risk factors for LTBI than TST whereas TST was significantly more closely associated with BCG vaccination than QuantiFERON^®^-TB Gold in-tube [19]. Moreover, the odds of a positive QuantiFERON^®^-TB Gold in-tube result, but not TST result, increased with increasingly relevant prognostic risk factors for LTBI from born or resident in a high prevalence country to a history of active tuberculosis. In the second study, IGRAs were positive in 7 TST-negative patients with IMID and risk factors for TB infection [38].

The only gold standard for LTBI is the subsequent development of TB but the generation of such data requires large longitudinal clinical outcome studies to establish the prognostic value of positive test results. The only published prospective data to date is too small to be able to draw any conclusions, comprising follow-up of 7 RA patients with positive QuantiFERON^®^-TB Gold results, 4 of whom subsequently started anti-TNF agents, for between 6 and 30 months, but none developed active TB [25].

## 5 Does immunosuppression affect IFN-γ production or reliability of IGRAs?

The performance of each IGRA is assessed through the use of an internal control which measures the IFN-γ response against phytohaemagglutinin (PHA). A negative response against this mitogen in the context of negative responses against the *M. tuberculosis*-specific antigens renders the IGRA indeterminate. This positive control becomes especially important in testing persons with weakened cellular immunity. To date the indeterminate rate of QuantiFERON^®^-TB Gold and QuantiFERON^®^-TB Gold in-tube in patients on immunosuppressive therapy has been between 2% and 12% [19–22,25,26,38]. The reported indeterminate rate of ELISpot was 0% in two studies [24,32] and 5.8% in a third study [38], but the limited amount of available data precludes drawing firm conclusions.

The apparent robustness of the technical performance and diagnostic sensitivity of IGRAs in IMIDs on DMARDs contrasts with the suppression of lymphoproliferative responses and IFN-γ responses in 5-day ELISAs previously noted in these populations [27]. This may reflect the fact that IGRAs measure IFN-γ released ex vivo by effector T cells rather than T-cell proliferation or IFN-γ released from cells that have undergone proliferation [27–29].

## 6 Clinical performance of IGRAs in patients on anti-TNF therapy

Three studies to date report on the performance of IGRAs in patients already on anti-TNF agents. Neither corticosteroids nor DMARDs significantly affected the QFT-Gold in-tube response in patients with inflammatory rheumatic conditions, but the odds for a positive IFN-γ result were decreased in patients treated with TNFα inhibitors [19]. In a second study, the magnitude of the IFN-γ response measured by ELISpot also significantly decreased 14 weeks after the start of anti-TNF treatment [27]. In a third recent study using QFT-G, 2 patients with positive IGRA results at 12 months of adalimumab therapy developed active TB [39]. The performance of IGRAs during anti-TNF treatment therefore needs to be systematically assessed to determine whether these tests can be used, if required, for regular screening of patients on anti-TNF agents in high prevalence countries or after an exposure event. In addition, alternative cytokine read-outs should be assessed to see if they are more robust to TNF blockade [30–32].

## 7 Concluding remarks

Published evidence on the performance of IGRAs suggests that IGRAs maintain their diagnostic sensitivity for LTBI better than TST in patients with IMID, most of whom are on immunosuppressive DMARDs. Most of the available data to date is derived from QFT-Gold in-tube and the evidence-base for both this assay and T-SPOT.*TB* needs to be expanded to assess whether these new tests are definitely superior to TST in this important patient population. Also, next-generation IGRAs that incorporate new *M. tuberculosis*-specific antigens which significantly enhance diagnostic sensitivity without compromising specificity hold promise and should therefore be assessed in this population [33]. The lack of a gold standard for diagnosis of LTBI means that prospective longitudinal clinical outcome studies to determine the prognostic value of positive IGRA results for subsequent development of active TB in this population are urgently required.

In the meantime, some practical recommendations for screening for TB infection prior to TNF blockade can be made. Most importantly, active TB must first be excluded by history and chest radiograph. Screening for LTBI should include checking for a history of untreated or partially treated TB, risk-stratification for exposure to cases of active TB and searching for evidence of residual changes indicative of untreated prior TB infection on a chest radiograph (e.g. calcified granulomas, Gohn focus or complex, apical scarring). In addition, given the apparent diagnostic superiority of IGRAs over TST, one or the other of the new blood tests should be performed. However, given the very limited size of the evidence-base in support of IGRAs to date and the vulnerability of these patients to develop severe and disseminated forms of TB on TNF blockade, it may be prudent to perform TST in parallel with IGRA to maximise the diagnostic sensitivity of screening, at least until the IGRA evidence-base in this population has expanded sufficiently. Since some patients with LTBI may have false-negative IGRA results and true-positive TST results, while others will have true-positive IGRA results and false-negative TST results, a dual-testing strategy should enable physicians to offer all patients with LTBI preventative treatment. IGRA results in patients already taking anti-TNF agents currently remain uninterpretable.

## Conflict of interest

Professor Lalvani is named inventor for several patents underpinning T-cell-based diagnosis. The Lalvani ELISpot was commercialised by an Oxford University spin-out company (T-SPOT.*TB*^®^, Oxford Immunotec Ltd, Abingdon, UK) in which Oxford University and Professor Lalvani have minority shares of equity. Professor Lalvani acted as non-executive director to Oxford Immunotec from 2003–07.

## Take-home messages

• Testing for and treatment of latent tuberculosis infection (LTBI) should be targeted to high risk groups who are at increased risk of progression to active tuberculosis.
• Tumour necrosis factor alpha (TNFα) is a key cytokine in protective host immunity against *M. tuberculosis* infection. The risk of incident tuberculosis (TB) in patients latently infected with *M. tuberculosis* increases substantially, from 4 to 5 fold over baseline in the case of infliximab, after initiation of anti-TNF therapy.
• TST is an inadequate tool for screening patients for LTBI before commencing anti-TNFα agents because of poor specificity in BCG-vaccinated individuals and poor sensitivity in patients with immune-mediated inflammatory diseases (IMID) on conventional immunosuppressants e.g. methotrexate and steroids.
• T-cell interferon-gamma release assays (IGRAs) detect interferon-gamma (IFN-γ) released ex vivo from pre-sensitised *M. tuberculosis*-specific T cells in response to selected proteins which, unlike PPD, are highly specific for *M. tuberculosis*. Extensive published literature suggests that IGRA are a more specific and probably a more sensitive test for diagnosis of *M. tuberculosis* infection than the TST in immunocompetent persons.
• The limited available published data in patients with IMID on disease modifying antirheumatic drugs (DMARDs) who are candidates for TNF blockade shows that IGRA results agree poorly with TST results and are more closely associated with the presence of risk factors for LTBI than TST, suggesting that IGRAs maintain diagnostic sensitivity for LTBI better than TST in this key population. However, preliminary data suggests that in patients who are already on anti-TNF treatment the IFN-γ response is significantly reduced and negative IGRA results should therefore not be interpreted as implying absence of infection.
• IGRAs are a promising adjunct for screening patients with IMID prior to TNF blockade, but more data are required to validate their precise role in this setting.

## Figures and Tables

**Table 1:** 

Study, reference	Assay	No. patients with IMID	Main findings
Bocchino et al. [38]	QFT-IT T-SPOT.*TB*	66	8/15 patients with IMID and risk factors for TB infection were positive to TST, T-SPOT.*TB* and QFT-IT. The remaining 7 were negative to TST but positive to either T-SPOT.*TB* and/or QFT-IT. 35/51 patients with IMID without risk factors for TB infection were negative to TST, T-SPOT.*TB* and QFT-IT.
Ponce de Leon et al. [22]	QFT-IT	101	Proportion of TST-positive results was significantly less in patients with IMID than in healthy controls (27/101 (26.7%) vs 61/93 (65.6%) *P* < 0.001) and size TST response significantly less (mean 3.73 vs 11.0 mm *P* < 0.001) Proportion TST-positive results in patients with IMID was 41% of healthy controls, significantly lower than proportion QFT-IT-positive results in patients with IMIDs was 75% of healthy controls (P = 0.008)
			Difference in proportion TST-positive & QFT-IT-positive was significant in patients with IMID (*P* = 0.013) but not in healthy controls (*P* = 0.45)
			Agreement between QFT-IT & TST results was poor in patients with IMID (agreement 70% *k* = 0.37)
			Agreement between QFT-IT & TST results was good in healthy controls (*n* = 93, agreement 83% *k* = 0.64)
Vassilopoulos et al. [24]	T-SPOT.*TB*	70	Agreement between TST and T-SPOT.*TB* results was moderate (72.8%)
			On multivariate analysis BCG was associated with TST-positive ELISpot-negative results (*P* = 0.01)
			On multivariate analysis steroid use was associated with TST-negative ELISpot-positive results (*P* = 0.04)
Matulis et al. [19]	QFT-IT	142	QFT-IT associated more closely with presence risk factors for LTBI than TST (OR 23.8 (95% CI 5.14 to 110 vs OR 2.77 (95% CI 1.22 to 6.27 *P* = 0.009).
			Odds QFT-IT-positive result increased with increasingly relevant markers of LTBI risk factors.
			QFT-IT associated less closely with BCG than TST (OR 0.47 (95% CI 0.15 to 1.47) vs OR 2.44 (95% CI 0.74 to 8.01) *P* = 0.025)
			Agreement between QFT-IT & TST results was low (agreement 64% *k* = 0.17 (95% CI 0.02 to 0.32))
Cobanoglu et al. [20]	QFT-IT	68	Agreement between TST & QFT-IT results in patients with IMID was poor (agreement 47% & 55% *k* = 0.18)
			Agreement between TST & QFT-IT results in healthy controls (*n* = 38) was poor (agreement 64% *k* = − 0.54)
Takahashi et al. [21]	QFT-G	14	Agreement between conventional diagnosis for LTBI (TST, chest radiography & medical history) and QFT-G results was moderate (64.3%).
Sellam et al. [23]	T-SPOT.*TB*	7	Agreement between TST and T-SPOT.*TB* results in patients with IMID with confirmed LTBI based on previous primo-infection, previous TB with inadequate treatment or TB lesions on chest radiograph (independent of TST) was moderate (71%).
Pratt et al. [25]	QFT-G	101	7/101 (7%) patients with rheumatoid arthritis were QFT-G-positive. 4/7 were started on anti-TNF treatment. No cases of *M. tuberculosis* reactivation or de novo infection in 98 patients within 6 to 30 months following initiation anti-TNF treatment.

## Glossary

BCG: bacille Calmette–Guérin
DMARDs: disease modifying antirheumatic drugs
ELISA: enzyme-linked immunosorbent assay
ELISpot: enzyme-linked immunospot
M. tuberculosis: Mycobacterium tuberculosis
LTBI: latent tuberculosis infection
PHA: phytohaemagglutinin
PPD: purified protein derivative
IGRA: T-cell interferon-gamma release assays
TNF: tumour necrosis factor
TST: tuberculin skin test
